# Beyond mass effect: An age-stratified analysis of CSF circulation disturbance in symptomatic intracranial arachnoid cysts

**DOI:** 10.1016/j.bas.2026.106159

**Published:** 2026-07-01

**Authors:** Maximilian Middelkamp, Benedikt Asey, Thomas Sauvigny, Lasse Dührsen, Sven Duda

**Affiliations:** Department of Neurosurgery, University Medical Center Hamburg‐Eppendorf, Hamburg, Germany

**Keywords:** Arachnoid cyst, Cerebrospinal fluid dynamics, Hydrocephalus, Pediatric neurosurgery, CSF circulation, Surgical outcomes

## Abstract

**Introduction:**

Symptomatic intracranial arachnoid cysts (AC) are commonly regarded as space-occupying lesions in which morbidity is attributed to mass effect and cyst volume. However, age-dependent differences in presentation suggest that disturbed cerebrospinal fluid (CSF) dynamics may substantially influence outcome, particularly in pediatric patients.

**Research question:**

We investigated whether markers of impaired CSF circulation are associated with postoperative morbidity and revision burden in symptomatic AC and whether these associations differ between pediatric and adult patients.

**Material and methods:**

This retrospective single-center cohort study included 30 patients who underwent surgery for symptomatic intracranial AC over 15 years. Patients were stratified into pediatric (<18 years) and adult groups. Clinical presentation, radiological markers of CSF disturbance (hydrocephalus, aqueductal stenosis, head circumference percentile), surgical course, reoperation rate, and functional outcomes (modified Rankin Scale [mRS], Karnofsky Performance Status [KPS], Lansky Play-Performance Scale (LPPS)) were analyzed.

**Results:**

Pediatric patients more frequently exhibited hydrocephalus and aqueductal stenosis. Postoperative hydrocephalus was strongly associated with increased reoperation frequency and worse functional outcome. Surgical burden correlated with worse outcome scales. Cyst volume reduction did not consistently correlate with clinical improvement. Younger age, increased head circumference, and neurenteric histology predicted salvage CSF diversion.

**Discussion and conclusion:**

Disturbed CSF dynamics are closely associated with morbidity and revision burden, particularly in pediatric patients. These findings support an age-dependent framework in which pediatric AC may primarily represent CSF circulation disorders, underscoring the importance of early recognition and targeted management.

## Highlights

• CSF disturbance predicts morbidity beyond cyst volume
• Pediatric cysts show higher hydrocephalus and revision burden
• Postoperative hydrocephalus drives reoperation frequency
• Surgical burden correlates with worse outcome scales
• Volume reduction does not predict functional improvement

## Abbreviations:

AC –: Arachnoid cyst
BBB –: Blood–brain barrier
CN –: Cranial nerve
CNS –: Central nervous system
CP –: Cystoperitoneal
CSF –: Cerebrospinal fluid
ETV –: Endoscopic third ventriculostomy
ICP –: Intracranial pressure
IQR –: Interquartile range
KPS –: Karnofsky Performance Status
mRS –: Modified Rankin Scale
MRI –: Magnetic resonance imaging
NKCC1 –: Na^+^–K^+^–2Cl^-^ cotransporter
OR –: Odds ratio
PFAC –: Posterior fossa arachnoid cyst
SEM –: Standard error of the mean
VP –: Ventriculoperitoneal

## 1 Introduction

Intracranial arachnoid cysts (AC) are benign cerebrospinal fluid (CSF)-containing lesions, accounting for approximately 1-2% of all intracranial mass lesions (Singh et al., 2023; Westermaier et al., 2012). These lesions are characterized by a split arachnoid membrane, absent traversing trabecular processes within the cyst, and thick collagen and hyperplastic arachnoid cells in the cyst wall (Ahmed and Cohen, 2023). They are increasingly detected incidentally with the widespread use of neuroimaging, with a reported prevalence of 2.6% in the pediatric population (Sarwar and Rocker, 2023). While the majority (>90%) of AC remain asymptomatic throughout life, symptomatic cysts may cause significant morbidity, traditionally attributed to local mass effect, mechanical compression of adjacent neural structures, and obstruction of CSF pathways, which may ultimately result in hydrocephalus (Singh et al., 2023; Westermaier et al., 2012; Ahmed and Cohen, 2023; Sarwar and Rocker, 2023; Hall et al., 2019).

Accordingly, surgical strategies have primarily focused on cyst decompression through fenestration, resection, or CSF diversion (Hall et al., 2019; Hayes et al., 2019; Jaradat et al., 2024). The mechanism of cyst enlargement remains incompletely understood. One-way slit valves have been identified in some cases, particularly in suprasellar and prepontine locations, where CSF pulsations during the cardiac cycle may drive progressive cyst expansion. The valve is typically located directly over the basilar artery, with cranially directed CSF flow increasing valve opening, while caudally directed flow pushes the slit against the artery, creating a one-way mechanism (Halani et al., 2013). Alternative theories include osmotic gradients between intra- and extracystic medium, primary malformation of the arachnoid membrane, and fluid hypersecretion by cyst wall lining cells (Basaldella et al., 2007). Molecular evidence supports the secretory hypothesis, with increased expression of the Na^+^-K^+^-2Cl^-^ cotransporter NKCC1 demonstrated in arachnoid cyst walls, suggesting active fluid secretion as a key mechanism of cyst filling (Helland et al., 2010).

However, clinical presentation differs markedly between pediatric and adult patients, suggesting that age-related variations in CSF physiology and cranial compliance may fundamentally influence disease pathophysiology. Children might present with signs of increased intracranial pressure, hydrocephalus, and progressive head enlargement, whereas adults frequently exhibit focal neurological deficits such as cranial nerve dysfunction or chronic headache (Singh et al., 2023; Ahmed and Cohen, 2023; Junger et al., 2022; Schmutzer-Sondergeld et al., 2024). Patients with symptomatic cysts are significantly younger and more likely to have associated hydrocephalus compared to those with asymptomatic lesions. Notably, obstructive hydrocephalus in the setting of AC has been observed almost exclusively in the pediatric population (Hall et al., 2019).

Age-related differences in intracranial compliance and cerebrospinal fluid (CSF) mechanisms are notable. CSF pressure-volume compensation varies with age. Resistance to CSF outflow rises sharply after 56 years, while younger individuals have higher CSF production and greater compensatory reserves (Czosnyka et al., 2001). The flexible pediatric skull allows volume adaptation until reserve is exceeded, risking hydrocephalus and intracranial hypertension (Czosnyka et al., 2001; Kahle et al., 2016). Intracranial pressure drops by about 0.69 mmHg each decade, and cerebrovascular stiffening reduces the brain's ability to manage volume changes as people age (Brasil et al., 2025; Kasprowicz et al., 2025). Production decreases in the elderly, choroid plexus perfusion drops, but its volume rises (May et al., 1990). The effective CSF flow declines, while regurgitant fraction increases (Eisma et al., 2023).

The relationship between AC and hydrocephalus is complex and bidirectional. Hydrocephalus is frequently found in midline and posterior fossa AC, while middle fossa lesions rarely produce hydrocephalus (Martinez-Lage et al., 2011). Some authors have proposed that AC may represent a localized form of hydrocephalus, with aberrant CSF dynamics playing a major pathogenic role in both cyst formation and associated ventriculomegaly (Martinez-Lage et al., 2011). Endoscopic exploration has revealed that ventricular abnormalities contributing to hydrocephalus—including foramen of Monro stenosis (86%) and aqueductal occlusion (71%)—may be more frequently associated with AC than previously recognized, suggesting a common developmental origin rather than a purely causal relationship (Rangel-Castilla et al., 2009). These findings support the hypothesis that some AC and associated hydrocephalus may share common pathogenic mechanisms related to disturbed CSF dynamics during development.

While numerous studies have evaluated surgical techniques, comparatively little attention has been paid to the role of CSF circulation disturbance as a determinant of postoperative morbidity and revision burden (Hall et al., 2019; Hayes et al., 2019; Jaradat et al., 2024). Reoperation rates vary widely across series, ranging from 14 to 34%, with significantly higher rates in young infants, particularly those under 1 year of age (Hall et al., 2019; Junger et al., 2022; Chan et al., 2021). We therefore hypothesized that markers of CSF circulation disturbance are more strongly associated with postoperative morbidity and reoperation burden than cyst volume itself, and that this association is particularly pronounced in pediatric patients.

## 2 Methods

### 2.1 Study design and data collection

This retrospective single-center cohort study included all patients who underwent neurosurgical procedures for intracranial AC at the Department of Neurosurgery, University Medical Center Hamburg-Eppendorf over a 15-year period. Surgical treatment was considered in patients with symptomatic AC causing neurological symptoms, hydrocephalus, progressive increase in head circumference, documented cyst enlargement, or significant mass effect. Patients treated for pineal cysts, Rathke cleft cysts, or tumor-associated cysts were excluded. To maintain a homogenous study cohort, all histologically confirmed neurenteric cysts were excluded from the final analysis. Clinical and radiological data were extracted from medical records, including demographics, presenting symptoms, imaging findings, surgical procedures, and follow-up outcomes.

### 2.2 Statistical analysis

Continuous variables are presented as mean ± standard deviation, unless otherwise specified. Categorical variables are presented as absolute and relative frequencies. Student's t-test and Fisher's exact test were used for group comparisons. Spearman correlation analysis assessed associations between continuous variables. Wilcoxon Signed-Rank-Test was used for analyzing volume measurements. Scatter Plots are shown as mean ± standard error of the mean. Boxplots display the median as a horizontal line within the box. The interquartile range (IQR), spanning from the 25th to the 75th percentile, is represented by the box itself. The whiskers extend to the last data point that falls within 1.5 times the IQR from the quartiles. Outliers are not shown in the plot. Due to missing data in some variables, the final analyzed sample size deviates from the original n = 30. To ensure full scientific transparency, all statistical analyses report the exact, effective sample size (n). A P value of less than 0.05 was considered as statistically significant. All analyses were performed using RStudio Version 2025.05.1 + 513. Data illustrations were performed using RStudio 2023.09.1, GraphPad Prism 10 and Microsoft PowerPoint.

## 3 Results

### 3.1 Cohort characteristics and age-dependent presentation

#### 3.1.1 Patient demographics

**Fig. 1:**
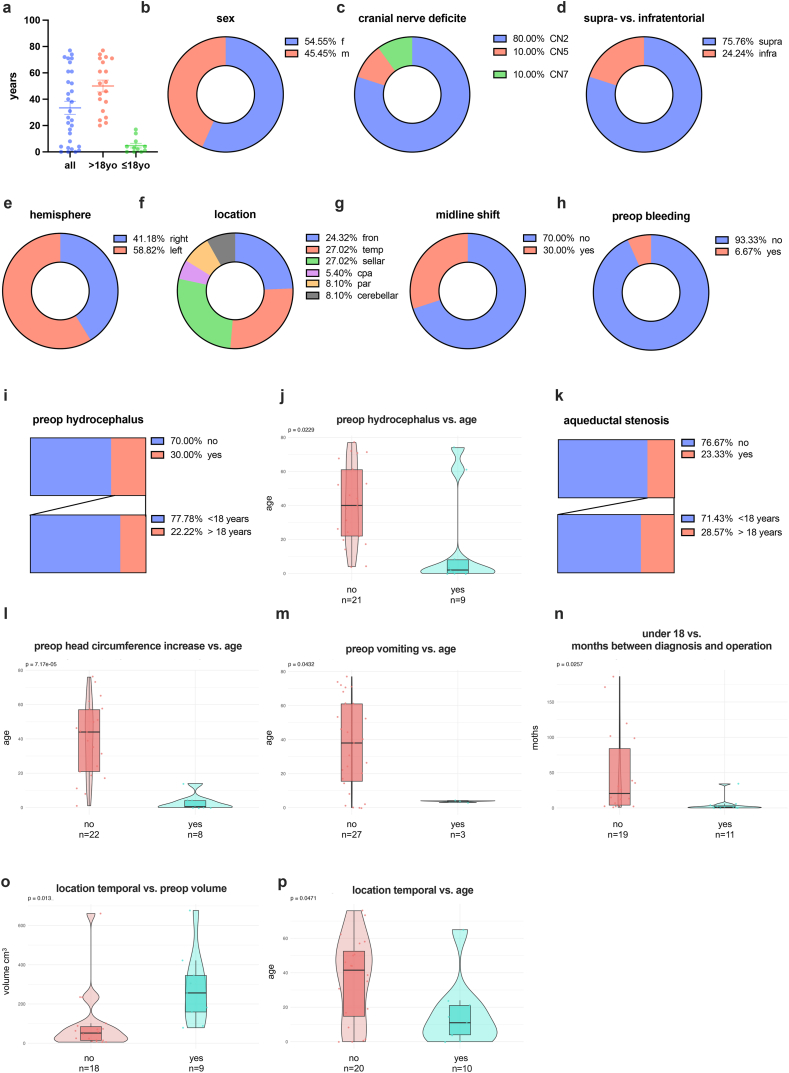
Cohort characteristics, anatomical distribution, and markers of CSF circulation disturbance. (a) Age distribution of the study cohort. (b) Sex distribution. (c) Distribution of cranial nerve deficits. (d) Supratentorial versus infratentorial cyst distribution. (e) Hemispheric distribution of cysts. (f) Detailed anatomical cyst locations. (g) Frequency of midline shift. (h) Frequency of preoperative intracystic hemorrhage. (i) Preoperative hydrocephalus frequency stratified by age group, demonstrating predominance in pediatric patients. (j) Age comparison between patients with and without preoperative hydrocephalus. (k) Aqueductal stenosis frequency stratified by age group. (l) Association between head circumference and patient age. (m) Age distribution in patients with and without preoperative vomiting. (n) between diagnosis and surgery stratified by age group. (o) Preoperative cyst volume stratified by temporal versus non-temporal location. (p) Age distribution stratified by temporal versus non-temporal cyst location.

Age at diagnosis for the entire cohort (n = 30) was 33.46 (range 0-77) years. Adult patients (≥18 years, n = 19) had a mean age of 50.05 (range 20-77) years, whereas pediatric patients (<18 years, n = 11) had a mean age of 4.81 (range 0-17 years) (Fig. 1a). Sex distribution showed slight female predominance (56.66%, n = 17 female; 43.33%, n = 13 male) (Fig. 1b).

#### 3.1.2 Clinical presentation patterns, anatomical distribution and radiological characteristics

Cranial nerve (CN) deficits were documented with CN2 involvement being most frequent (80.00%, n = 8 of 10 total CN deficits), followed by CN5 (10.00%, n = 1), and CN7 (10.00%, n = 1) Fig. 1c). Most cysts were located supratentorial (75.76%, n = 24), with the remainder infratentorial (24.24%, n = 6) (Fig. 1d). For non-midline cysts, hemispheric distribution was split between left (58.82%, n = 10) and right (41.18%, n = 7) (Fig. 1e). Among 37 recorded cyst locations, the most frequent sites were temporal (n = 10, 27.02%), sellar (n = 10, 27.02%), frontal (n = 9, 24.32%), parietal (n = 3, 8.10%) and supracerebellar (n = 3, 8.10%) and cerebellopontine angle (n = 2, 5.40%) (Fig. 1f). For large cysts, multiple anatomical locations were documented. Midline shift was observed in 30.00% (n = 10 of 30) of cases (Fig. 1g), indicating significant mass effect in a subset of patients. Preoperative bleeding into the cyst was documented in 6.67% (n = 2) (Fig. 1h).

#### 3.1.3 Markers of CSF circulation disturbance

Preoperative hydrocephalus was present in 30.00% (n = 9) of all patients and occurred predominantly in pediatric cases (Fig. 1i). Among those with hydrocephalus, 77.78% (n = 7) were under 18 years old, and 22.22% (n = 2) were adults. Patients with hydrocephalus were significantly younger (mean age 16.55 ± 29.17 years, n = 9) compared to those without hydrocephalus (40.71 ± 23.40 years, n = 21; p = 0.0229) (Fig. 1j). Aqueductal stenosis was found in 23.33% (n = 7 of 30) of cases and was more common in pediatric patients (71.43%, n = 5 of 7) (Fig. 1k). Increased head circumference was strongly associated with younger age (p = 7.17e−05; Fig. 1l). Head circumference data was available for all pediatric patients, with a mean percentile of 92.18 ± 10.75. Vomiting occurred exclusively in very young patients (mean age 3.67 ± 0.57 years, n = 3) compared to those without vomiting (36.77 ± 26.65 years, n = 27; p = 0.0432) (Fig. 1m). The interval between diagnosis and surgery was significantly shorter in pediatric patients (4.54 ± 9.95 months, n = 11) compared to adults (48.11 ± 60.27 months, n = 18; p = 0.0257) (Fig. 1n), and positively correlated with age (Spearman r = 0.60, p = 0.0005, n = 29; Supplementary Fig. 1a).

#### 3.1.4 Cyst location and volume relationships

Temporal cyst location was associated with greater lesion volumes (267.13 ± 189.86 cm^3^, n = 9) compared to other locations (92.97 ± 158.60 cm^3^, n = 18; p = 0.013) (Fig. 1o) and occurred more frequently in younger patients (mean age 15.89 ± 20.36 years, n = 10) versus other locations (35.60 ± 24.84 years, n = 20; p = 0.0471) (Fig. 1p). Preoperative cyst volume negatively correlated with age (Spearman r = −0.44, p = 0.022, n = 27; Supplementary Fig. 1b).

### 3.2 Surgical course and determinants of reoperation

#### 3.2.1 Baseline parameters and surgical indications

**Fig. 2:**
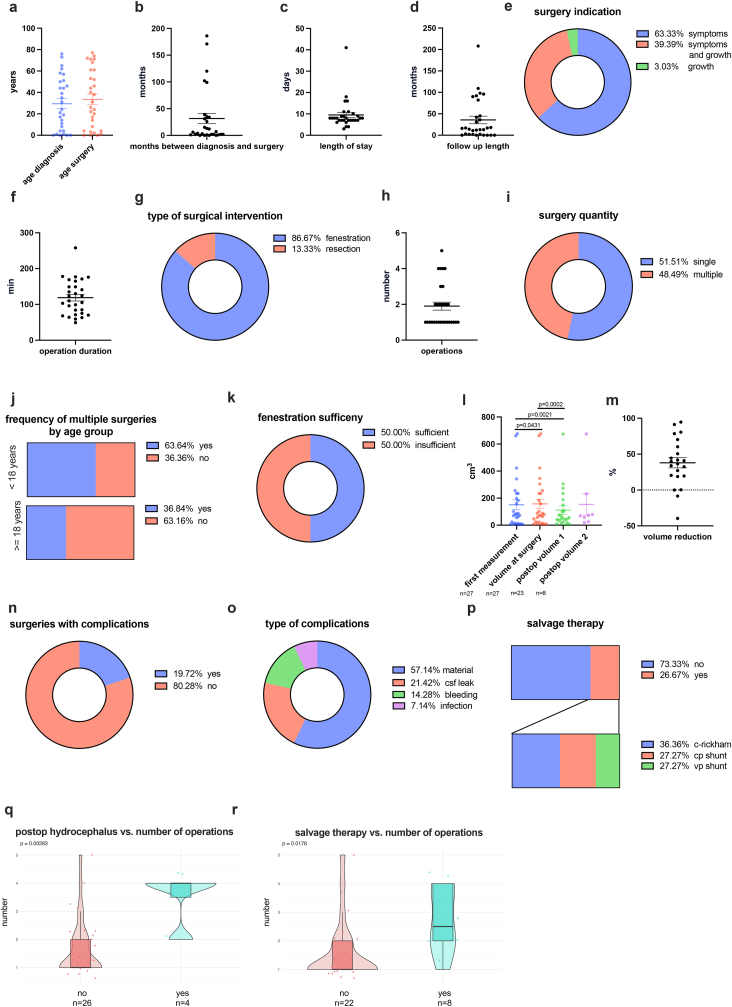
Surgical course, complications, and determinants of reoperation. (a) Age at diagnosis versus age at surgery. (b) Interval between diagnosis and surgical intervention. (c) Hospital stay duration. (d) Follow-up duration. (e) Surgical indications. (f) Operation duration. (g) Distribution of primary surgical procedures. (h) Number of operations per patient. (i) Distribution of single versus multiple surgeries. (j) Frequency of multiple surgeries stratified by age group. (k) Adequacy of fenestration as definitive treatment. (l) Serial cyst volume measurements from diagnosis through postoperative follow-up. (m) Relative cyst volume reduction. (n) Overall complication rate. (o) Distribution of complication types. (p) Salvage therapy requirement and modalities employed. (q) Number of surgeries stratified by postoperative hydrocephalus status. (r) Number of surgeries stratified by salvage therapy requirement.

The mean age at diagnosis was 29.48 ± 24.53 years (n = 30), increasing to 33.46 ± 26.73 years at the time of surgery (Fig. 2a). The average duration between diagnosis and surgical intervention was 31.58 ± 51.09 months (n = 29) (Fig. 2b). Patients had a mean hospital stay of 9.5 ± 6.70 days (n = 30) (Fig. 2c) and a mean follow-up period of 35.63 ± 47.32 months (n = 30) (Fig. 2d). Surgery was performed for symptomatic cysts (63.33%, n = 19), cysts that were symptomatic with documented growth on serial imaging (39.39%, n = 10), and cyst growth alone (3.03%, n = 1) (Fig. 2e). The average operation duration was 118.82 ± 48.13 min (n = 29) (Fig. 2f).

#### 3.2.2 Primary procedures and revision patterns

Fenestration was the predominant primary procedure (86.67%, n = 26), while cyst resection was performed in 13.33% (n = 4) (Fig. 2g). The average number of operations per patient was 1.90 ± 1.19 (n = 30) (Fig. 2h). Nearly half of patients (48.49%, n = 14) required multiple surgeries due to insufficient symptom relief, while 51.51% (n = 16) underwent a single surgical intervention (Fig. 2i). Multiple surgeries were more frequent in pediatric patients (63.64%, n = 7) compared to adults (36.84%, n = 7) (Fig. 2j). When analyzing primary cyst fenestrations alone, revision surgery was required in 50.00% (n = 13 of 26) of cases (Fig. 2k).

#### 3.2.3 Volumetric changes

A significant increase in lesion volume was observed between the initial preoperative (158,82 cm^3^ ± 182.37 cm^3^, n = 27) assessment and the time of surgical indication (165,52 cm^3^ ± 183.01 cm^3^ (n = 27, p = 0.0431). Postoperatively, lesion volume decreased significantly (110,95 cm^3^ ± 146.82 cm^3^, n = 23) compared with both the initial preoperative measurement (p = 0.0021) and the volume at the time of surgical indication (p < 0.0002). No statistically significant differences were observed at the second postoperative follow-up (153,87 cm^3^ ± 206.40 cm^3^, n = 8) (Fig. 2l). The mean relative volume reduction was 37.94% (n = 22) (Fig. 2m).

#### 3.2.4 Complications and salvage therapy

Complications were recorded in 19.72% (n = 14) of a total of 71 surgeries performed during the follow-up period (Fig. 2n). The most common complications were material-related (57.14%, n = 8), followed by cerebrospinal fluid (CSF) leak (21.42%, n = 3), bleeding (14.28%, n = 2), infection (7.14%, n = 1) (Fig. 2o). Salvage therapy was required in 26.67% of all patients (n = 8) (Fig. 2p), with cystic Rickham reservoir (44.44%, n = 4), cystoperitoneal (CP) shunt (33.33%, n = 3), ventriculoperitoneal (VP) shunt (22.22%, n = 2) employed as rescue modalities, including one patient who received a conversion from cystic Rickham to cystoperitoneal shunt. Postoperative hydrocephalus was strongly associated with increased number of surgeries (3.5 ± 1.00, n = 4 vs. 1.65 ± 1.05, n = 26; p = 0.0028) (Fig. 2q). Patients receiving salvage therapy also underwent significantly more surgeries (2.75 ± 1.16, n = 8 vs. 1.59 ± 1.09, n = 22; p = 0.0178) (Fig. 2r).

### 3.3 Functional outcome and impact of CSF disturbance

#### 3.3.1 Functional outcome over time

**Fig. 3:**
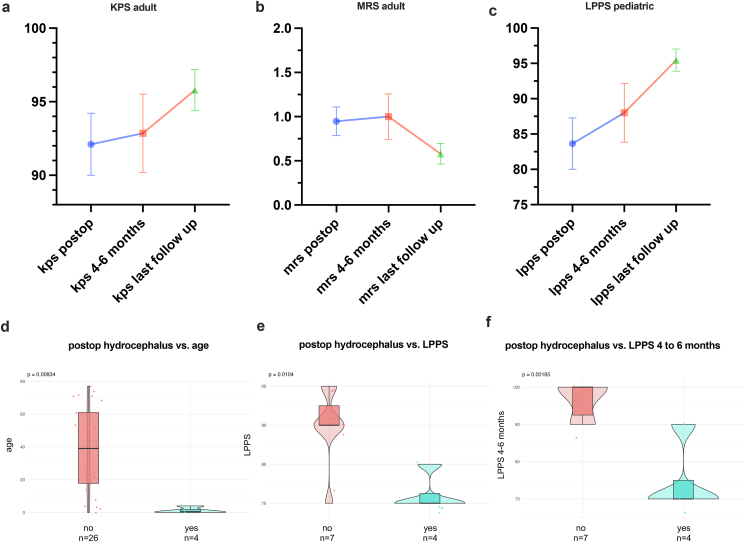
Functional outcomes and impact of CSF disturbance. (a) Karnofsky Performance Status over time from postoperative period through final follow-up. (b) Modified Rankin Scale over time from postoperative period through final follow-up. (c) Lanksy Play Performance Scale over time from postoperative period through final follow-up. (d) Age comparison between patients with and without postoperative hydrocephalus. (f) Follow-up duration stratified by postoperative hydrocephalus status. (e) LPPS comparison between patients with and without postoperative hydrocephalus. (f) LPPS after 4 to 6 months comparison between patients with and without postoperative hydrocephalus.

Functional outcome improved during the follow-up period. Postoperatively, the mean Karnofsky Performance Status (KPS) for adults was 92.10% ± 8.93 (n = 19), increasing to 92.85% ± 9.58 (n = 14) at 4–6 months and 95.78% ± 5.90 (n = 19) at final follow-up (Fig. 3a). The postoperative modified Rankin Scale (mRS) was 0.94 ± 0.68 (n = 19), increasing to 1.00 ± 0.92 (n = 14) at 4–6 months and 0.57 ± 0.49 (n = 19) at final follow-up (Fig. 3b). The postoperative Lansky Play-Performance Scale for pediatric patients (LPPS) was 83.63 ± 11.64 (n = 11), increasing to 88.00 ± 12.48 (n = 10) at 4–6 months and 95.45 ± 4.97 (n = 11) at final follow-up (Fig. 3c).

#### 3.3.2 Relationship between age and persistent postoperative hydrocephalus

Patients with persistent hydrocephalus were significantly younger (1.25 ± 1.89 years, n = 4) than those without (38.42 ± 25.79 years, n = 26; p = 0.0083) (Fig. 3d). Patients with persistent postoperative hydrocephalus had a significant lower LPPS after surgery (72.50 ± 5.00, n = 4 vs. 90.00 ± 10.00, n = 7, p = 0.0104) (Fig. 3e) and after 4 to 6 months (75.00 ± 10.00, n = 4 vs. 96.66 ± 5.16, n = 6, p = 0.0018) (Fig. 3f).

#### 3.3.3 Correlation analyses of surgical and follow-up parameters

Supplementary correlation analyses demonstrated that LPPS after 4 to 6 months negatively correlated with the number of surgeries (r = −0.70, p = 0.024, n = 10) (Supplementary Fig. 1c) and follow up length positively correlated with number of surgeries (r = 0.42, p = 0.021, n = 30) (Supplementary Fig. 1d). The postop LPPS negatively correlated with the follow up length (r = −0.64, p = 0.0340, n = 11) (Supplementary Fig. 1e). The postop mRS positively correlated with the follow up length (r = 0.67, p = 0.0017, n = 19) (Supplementary Fig. 1f).

## 4 Discussion

### 4.1 Age-dependent CSF disturbance: a pathophysiological framework

The age-stratified differences observed in our cohort align with emerging concepts in CSF physiology and intracranial compliance. Children with AC more frequently exhibited hydrocephalus, aqueductal stenosis, and macrocephaly, supporting a model in which pediatric AC may act predominantly as modulators of CSF dynamics rather than purely space-occupying lesions. This conceptual framework is supported by several lines of evidence from the literature. First, the compliant pediatric skull allows compensatory volume adaptation through cranial expansion until CSF compensatory reserve is exceeded (Schmutzer-Sondergeld et al., 2024; Kahle et al., 2016). Once compensatory mechanisms are exhausted, global intracranial hypertension and hydrocephalus may develop. Second, CSF dynamics demonstrate significant age-dependent variation. CSF flow and pulsatility decrease significantly with age from childhood through adulthood, while resistance to CSF outflow increases nonlinearly, particularly after age 56 years. (Czosnyka et al., 2001; Eisma et al., 2023; Sahoo et al., 2024). These physiological changes may explain why adult patients in our cohort more commonly presented with focal neurological deficits rather than signs of global CSF disturbance. The adult cranium's limited compliance restricts compensatory expansion, potentially channeling pathophysiology toward localized mass effect rather than diffuse CSF circulation disturbance. Third, the bidirectional relationship between AC and hydrocephalus suggests shared pathogenic mechanisms. Our findings are consistent with the concept proposed by Martínez-Lage et al. that, in selected patients, AC may represent a localized manifestation of disturbed CSF circulation rather than a purely space-occupying lesion (Martinez-Lage et al., 2011). The frequent coexistence of ventricular abnormalities such as foramen of Monro stenosis and aqueductal obstruction reported in endoscopic studies further supports the notion that AC and hydrocephalus may share common developmental and pathophysiological mechanisms (Rangel-Castilla et al., 2009).

In our cohort, aqueductal stenosis was more frequent in pediatric patients, and the interval between diagnosis and surgery was significantly shorter in children, suggesting more acute decompensation when CSF pathways are compromised. However, as our findings are based solely on radiological and clinical signs suggestive of disturbed CSF circulation, future studies incorporating direct assessment of CSF hydrodynamics, including flow-sensitive MRI techniques, are needed not only to clarify the underlying pathophysiological mechanisms but also to determine how these findings can be integrated into patient stratification and surgical decision-making.

### 4.2 Hydrocephalus and surgical burden: clinical implications

Postoperative hydrocephalus emerged as the strongest predictor of poor outcome in our cohort. Patients with persistent hydrocephalus required significantly more surgeries and demonstrated worse functional outcomes both early and at 4-6 months follow-up. This finding has important clinical implications for surgical planning and postoperative monitoring. The literature supports a high reoperation burden in pediatric AC patients, particularly in young infants. Jünger et al. reported reoperation rates of 33.3% for first revisions and 12.7% for second revisions, with significantly higher rates in children under 1 year (Junger et al., 2022). Similarly, Schmutzer-Sondergeld et al. identified age under 6 years, preoperative ventricular expansion, and paresis as significant risk factors for recurrence. Our findings extend these observations by demonstrating that postoperative hydrocephalus—rather than preoperative cyst characteristics—is the critical determinant of revision burden. The need for salvage CSF diversion in 26.66% of our cohort underscores the inadequacy of cyst fenestration alone in patients with underlying CSF circulation disturbance. When surgery is indicated, the presence of hydrocephalus, cyst location, and patient age should all be considered to optimize outcomes and reduce complications (Pesaresi et al., 2024). Beyond its impact on revision frequency, persistent postoperative hydrocephalus emerged as the principal determinant of functional outcome. These patients demonstrated significantly worse outcome scores (mRS, KPS; LPPS) both in the early postoperative phase and at 4–6 months follow-up, indicating sustained functional impairment rather than transient perioperative morbidity. Importantly, the number of surgical interventions correlated positively with worse outcome scales, suggesting that revision burden reflects ongoing CSF circulatory failure rather than being an isolated procedural consequence. In contrast, cyst volume reduction did not correlate with functional improvement. This dissociation reinforces the concept that restoration of CSF flow dynamics, rather than mechanical decompression alone, represents the critical determinant of neurological recovery. Functional disability in this context appears to arise from persistent disturbance of global CSF dynamics, particularly in younger patients with limited compensatory reserve once decompensation occurs.

### 4.3 Cyst volume reduction: necessary but not sufficient

Although cyst size influenced clinical presentation, with larger volumes associated with younger age and vomiting, statistically significant volumetric reduction did not consistently correlate with clinical improvement. This observation aligns with studies demonstrating a dissociation between radiological and clinical outcomes following arachnoid cyst surgery, which show that postoperative cyst volume reduction does not reliably correlate with clinical improvement and suggest that cyst size alone is an insufficient surrogate for treatment success (Helland and Wester, 2007; Pitsika and Sgouros, 2019; Rabiei et al., 2016, 2018; Tamimi et al., 2021). In a pediatric cohort with 8.6-year follow-up, Rabiei et al. found no association between radiological cyst volume reduction and clinical improvement, supporting a restrictive attitude toward surgery in the absence of objectively verified symptoms or CSF pathway obstruction (Rabiei et al., 2016). Consistent with this hypothesis, postoperative hydrocephalus, rather than residual cyst volume was associated with poorer functional outcomes, underscoring the limited value of postoperative cyst size as a surrogate marker of outcome.

### 4.4 Surgical strategy: fenestration versus shunting

The optimal surgical approach for symptomatic AC remains controversial. Recent literature suggests that endoscopic fenestration offers superior outcomes compared to cystoperitoneal shunting, with higher improvement rates (81.8% vs. 50%) and lower complication rates (45.5% vs. 65%) (Jaradat et al., 2024). Multiple fenestrations during endoscopic surgery significantly reduce the need for secondary procedures (5.3% vs. 36.1%, p < 0.001) (Guler et al., 2023). Meta-analyses support neuroendoscopic fenestration as the optimal initial procedure for middle cranial fossa arachnoid cysts, with lower long-term complication rates compared to microsurgical approaches (Chen et al., 2016). Oertel et al. reported an overall clinical success rate of 90% with endoscopic techniques, though temporobasal cysts were the most difficult to treat with lowest clinical success (81%), highest recurrence (19%), and highest complication rate (24%). Ventriculocystostomy and ventriculocystocisternostomy achieved the highest success rates at 100%. Recurrences can occur many years after the initial procedure, necessitating long-term follow-up (Oertel et al., 2010). However, these recommendations may not apply uniformly to patients with significant CSF circulation disturbance. In our cohort, 30.30% ultimately required salvage CSF diversion despite initial fenestration attempts. Martínez-Lage et al. emphasized that in patients with coexistent hydrocephalus and arachnoid cysts, the ideal approach should address both pathologies simultaneously (Martinez-Lage et al., 2011).

### 4.5 Age-specific management considerations

The natural history of AC supports selective intervention. Hall et al. demonstrated that asymptomatic AC have a low rate of enlargement on follow-up imaging (99.3% remained stable or reduced), supporting a conservative approach without routine imaging in asymptomatic cases. However, serial imaging and surgery may still be indicated in asymptomatic patients at risk of obstructive hydrocephalus, which was observed only in the pediatric population (Hall et al., 2019).

Our findings support an age-stratified approach to surgical management of AC. In pediatric patients, assessment should focus on markers of CSF circulation disturbance: hydrocephalus, aqueduct patency, head circumference percentile, and signs of increased intracranial pressure. The presence of these markers should prompt consideration of more aggressive CSF diversion strategies, potentially including endoscopic third ventriculostomy or ventriculoperitoneal shunting in addition to cyst fenestration. Close postoperative monitoring for hydrocephalus is critical, as persistent hydrocephalus strongly predicts revision burden and functional disability. The significantly shorter interval between diagnosis and surgery in pediatric patients suggests more rapid decompensation, necessitating expedited surgical planning when symptoms develop. This age-dependent pattern is further supported by the recent systematic review and meta-analysis by Jenkins et al., which identified younger age as an independent predictor of reoperation risk irrespective of surgical technique. These findings reinforce the concept that age-related factors substantially influence disease behavior and treatment response in pediatric arachnoid cysts (Jenkins et al., 2026). In adult patients, focal mass effect appears to predominate over global CSF disturbance. Fenestration alone may be more successful in this population. The age-related decrease in intracranial compliance may paradoxically protect against global CSF circulation disturbance while increasing susceptibility to focal neurological deficits (Brasil et al., 2025; Kasprowicz et al., 2025).

### 4.6 Genetic and developmental underpinnings of arachnoid cysts

Emerging evidence suggests that arachnoid cysts may, in selected patients, represent part of a broader developmental or genetic disorder rather than an isolated structural lesion. A large-scale exome analysis of 617 trios demonstrated a significant enrichment of damaging de novo variants in patients with arachnoid cysts, converging on chromatin modifiers and midgestational transcriptional networks essential for neural and meningeal development (Kundishora et al., 2023). Familial forms have been reported, and arachnoid cysts occur with increased frequency in multiple Mendelian syndromes, including Down syndrome, schizencephaly, neurofibromatosis, and autosomal dominant polycystic kidney disease (ADPKD) (Ahmed and Cohen, 2023; Qureshi et al., 2023). In ADPKD specifically, arachnoid cysts were found in 8.1% of patients versus 0.8% of matched controls, with PKD1 mutations conferring a fivefold increased risk (Shigemori et al., 2021). Notably, pathogenic GABRG2 loss-of-function variants have been associated with temporal arachnoid cysts in addition to their established role in epilepsy syndromes, further supporting a genetic contribution to cyst formation in specific brain regions (Schievink et al., 1995). These associations with alterations in neurodevelopment, ciliary function, and CSF regulation may provide a biological explanation for why some pediatric patients experience persistent CSF circulation disturbances or require revision procedures despite technically successful cyst fenestration.

### 4.7 Limitations

The relatively small cohort size, particularly in the pediatric subgroup, limits the statistical power of subgroup analysis and necessitates cautious interpretations of the observed associations. Furthermore, conclusions are based on radiological signs of CSF disturbance rather than direct of CSF hydrodynamics. Additional limitations include the heterogeneity of cyst locations and surgical approaches, and potential selection bias inherent to the retrospective design. Future multicenter prospective studies incorporating advanced CSF flow imaging and direct assessment of CSF dynamics are needed to validate these findings and further refine age-specific treatment strategies.

## 5 Conclusions

Markers suggestive of disturbed CSF dynamics were associated with increased postoperative morbidity and revision burden in pediatric patients with symptomatic intracranial arachnoid cysts, supporting the concept that age-dependent differences in CSF physiology may contribute to disease presentation and treatment response. Persistent hydrocephalus was associated with poorer functional outcomes and increased surgical burden, highlighting the importance of early recognition and management of CSF circulation disturbances. Collectively, these findings support a more physiology-oriented perspective on symptomatic arachnoid cysts and underscore the need for future studies incorporating direct assessment of CSF hydrodynamics to refine age-specific management strategies.

## Ethics approval and consent to participate

This study was performed in line with the principles of the Declaration of Helsinki. All research involving human participants adhered to ethical standards.

## Consent for publication

Not applicable.

## Availability of data and materials

Not applicable.

## Authors' contributions

Maximilian Middelkamp: Conceptualization, Project administration, Data curation, Formal analysis, Investigation, Methodology, Software, Validation, Visualization, Writing – original draft, Writing – review and editing.

Benedikt Asey: Conceptualization, Data curation, Investigation, Validation, Writing – original draft.

Thomas Sauvigny: Methodology, Data curation, Investigation, Validation, Writing – review and editing.

Lasse Dührsen: Methodology, Data curation, Investigation, Validation, Writing – review and editing.

Sven Duda: Conceptualization, Project administration, Data curation, Formal analysis, Investigation, Methodology, Validation, Visualization, Supervision, Writing – original draft, Writing – review and editing.

All authors have read and agreed to the published version of the manuscript.

## Declaration of generative AI in scientific writing

During the preparation of this work the authors used ChatGPT 5.2 for language improvement and OpenEvidence for additional literature research. After using these tools, the authors reviewed and edited the content as needed and take full responsibility for the content of the published article.

## Funding

The authors declare that no funds, grants, or other support were received during the preparation of this manuscript.

## Competing interests

The authors declare that they have no competing interests.

## Appendix A Supplementary data

Supplementary Figure 1. **Correlation analyses and predictors of salvage CSF diversion**. (a) Correlation between interval from diagnosis to surgery and patient age. (b) Correlation between preoperative cyst volume and patient age. (c) Correlation between LPPS at 4 to 6 months and number of surgeries. (d) Correlation between follow up length in months and number of surgeries. (e) Correlation between postop LPPS and follow up length in months. (f) Correlation of postop mRS and follow up length in months.Multimedia Component. 1
