# Supplementary figures and images for: Beyond mass effect: An age-stratified analysis of CSF circulation disturbance in symptomatic intracranial arachnoid cysts
Source: Brain Spine. 2026 Jul 1;6:106159. doi: 10.1016/j.bas.2026.106159 (PMC13348192; doi:10.1016/j.bas.2026.106159)

# Supplementary Figure 1

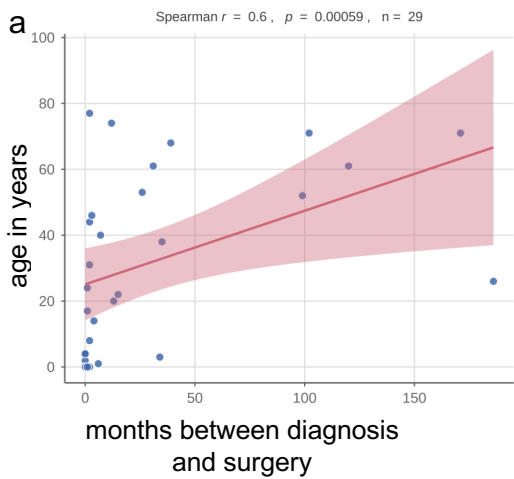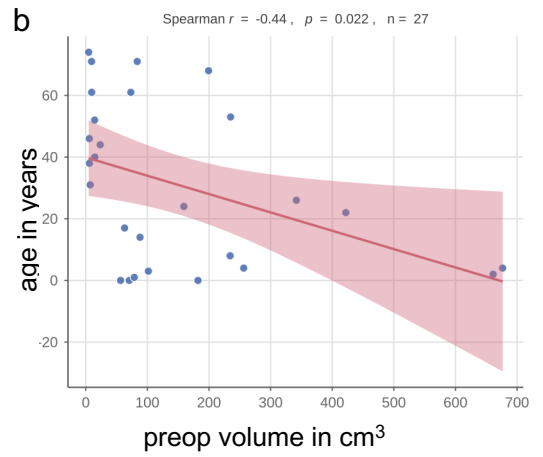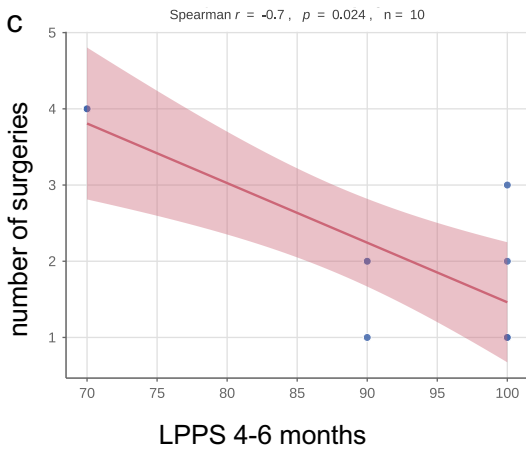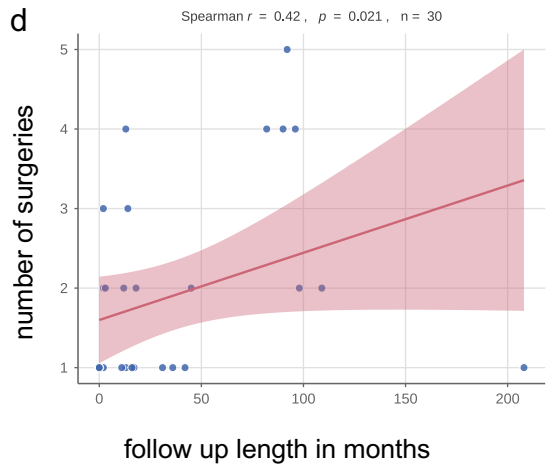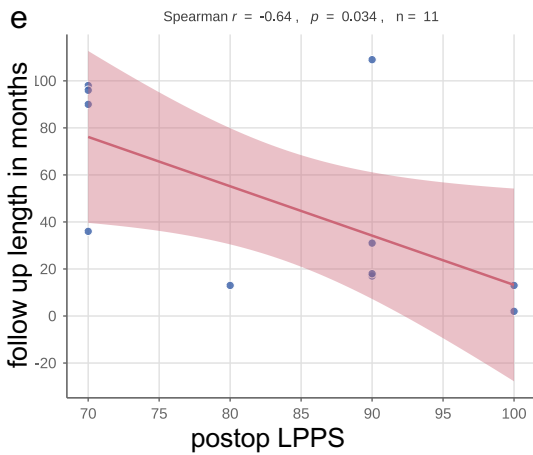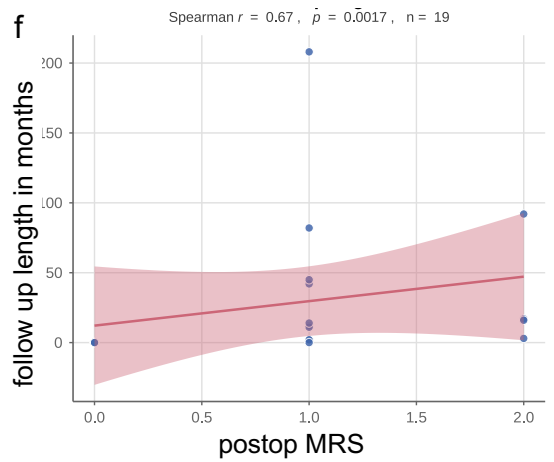

Supplement: Supplementary file 1 — Supplementary Figure 1. Correlation analyses and predictors of salvage CSF diversion. (a) Correlation between interval from diagnosis to surgery and patient age. (b) Correlation between preoperative cyst volume and patient age. (c) Correlation between LPPS at 4 to 6 months and number of surgeries. (d) Correlation between follow up length in months and number of surgeries. (e) Correlation between postop LPPS and follow up length in months. (f) Correlation of postop mRS and follow up length in months.Multimedia Component. 1 [file mmc1.pdf]
